# Luminescent Materials for Volumetric Three-Dimensional Displays Based on Photoactivated Phosphorescence

**DOI:** 10.3390/polym15092004

**Published:** 2023-04-24

**Authors:** Yuhan Gu, Shigang Wan, Qing Liu, Changqing Ye

**Affiliations:** 1 School of Materials Science and Engineering, Suzhou University of Science and Technology, Suzhou 215009, China; 2 School of Chemistry and Life Sciences, Suzhou University of Science and Technology, Suzhou 215009, China

**Keywords:** 3D display, photoactivation, phosphorescence, phthalocyanine, photochemical deoxygenation

## Abstract

True three-dimensional (3D) displays are the best display technologies and their breakthrough is primarily due to advancements in display media. In this paper, we propose two luminescent materials for a static volumetric 3D display based on photoactivated phosphorescence. The luminescent materials include (1) dimethyl sulfoxide (DMSO)/1-methyl-2-pyrrolidinone (NMP) or tetramethylene sulfoxide (TMSO) as the solvent and photochemically-deoxygenating reagent; (2) a metal phthalocyanine complex as the sensitizer; (3) a phosphorescent platinum complex as the emitter. The metal phthalocyanine complex, PdPrPc (PdBuPc), absorbs the light beam of 635 nm and the solvent scavenges the sensitized singlet oxygen. Light beams pass through a deoxygenated zone. The phosphorescent emitter, PtNI, absorbs the 440 nm light beam and phosphoresces only in the deoxygenated zone generated by the sensitizer. Phosphorescent voxels and high-contrast 3D images are well-defined at the intersection of 635 and 440 nm light beams.

## 1. Introduction

Volumetric three-dimensional (3D) displays can directly generate voxels in a 3D space and render volumetric graphics to viewers [1,2,3,4]. They have potential applications in medical imaging, education, and architectural and military modeling [5,6]. Volumetric 3D displays can be divided into swept and static volume displays with or without moving parts [4,7,8,9,10,11,12]. Among these 3D display technologies, beam-addressable static volume displays are attractive for their easy fabrication and simplified manipulation of voxels. In this display system, the key components are display mediums that determine the quality of reconstructed images. Some luminescent materials such as rare earth-doped materials [12,13,14,15,16,17], gaseous materials [18,19,20], dye-based composites [21], upconversion materials [12,22,23], and photoactivatable molecules [24,25,26,27] have been explored as display mediums for beam-addressable static volume displays.

Among these luminescent materials, rare earth upconversion materials have been extensively studied [28,29]. The voxels are defined by a process called two-step, two-frequency upconversion. Rare earth-doped glass was initially developed and used as a 3D display medium. Although 3D images are successfully constructed, interference during display may occur. Furthermore, obtaining scalable rare earth-doped glass is challenging [12,30]. Rare earth-doped polymers, which are scalable and less expensive, were later developed. However, due to the mismatch between refractive indexes, inherent scattering effects negatively influence image quality [15]. Developments in nanoscience and nanotechnology have created rare earth upconversion nanocrystals. By incorporating upconversion nanocrystals into the polymer matrix as a display medium, 3D images are constructed by computer-controlled near-infrared laser scanning [31]. Due to the low upconversion efficiency, high-power lasers are needed. Rare earth upconversion display materials are not ideal for practical applications. Organic dyes with two-photon absorption (TPA) properties can also be used as 3D display mediums. However, TPA in dyes involves the simultaneous absorption of two photons. This process requires two pulsed laser beams with spatial and temporal overlap [32]. Triplet–triplet annihilation (TTA)-based photon upconversion might be a promising candidate for 3D displays due to its high upconversion quantum yield at low excitation intensities [33]. Some challenges must be overcome before they can be used as luminescent materials in 3D displays, such as low efficiency in a solid matrix and oxygen-quenching of the excited triplet state.

In 2017, Lippert et al. reported on a volumetric 3D digital light display system based on the photoactivation of photochromic dye [26]. They designed a photochromic dye (*N*-phenyl spirolactam rhodamine) and a dichloromethane solution was used as the display medium. Upon excitation at 385 nm, the initially non-emissive state undergoes a ring-opening photoreaction. Then it becomes a fluorescent state with green-yellow emissions upon illumination with 525 nm light. The intersection of the 385- and 525-nm light beams defined the fluorescent voxels. The 3D images were generated by controlling the intersections of light beams from two projectors. In this 3D display system, the optical power densities of the excitation lights are 0.40 mW cm^−2^ for 385 nm and 2.0 mW cm^−2^ for 525 nm. It provides an exciting opportunity for the academic and industrial development of volumetric 3D displays. For this display system, due to the slight Stokes shift caused by the fluorescent nature of the photochromic dye, an optical filter was needed to visualize the 3D images. Fluorescent molecules incorporated into cryogels have also been used as display mediums for volumetric 3D displays. It is a challenge to increase cryogel size to fulfill their practical use [34].

Phosphorescence has a larger Stokes shift than fluorescence. Studies on room-temperature phosphorescence have a long history. Phosphorescent compounds have broad applications in bioimaging, encryption, sensing, and nonlinear optics [35,36,37,38]. Molecular oxygen can efficiently quench long-lived photoexcited triplet states of phosphorescent compounds [39]. Our recent studies revealed that photo-irradiation could competently and reversibly activate the long-lived phosphorescence of some metal–organic complexes in aerated liquid sulfoxide and cyclic urea solutions. We demonstrated these solvents’ deoxygenation mechanism and named the phenomenon photoactivated phosphorescence (PAP) [40,41]. This phenomenon was applied to the triplet–triplet annihilation photon-upconversion under aerated conditions [41] and the photo-patterning of a two-dimensional gel surface [42].

**Scheme 1:**
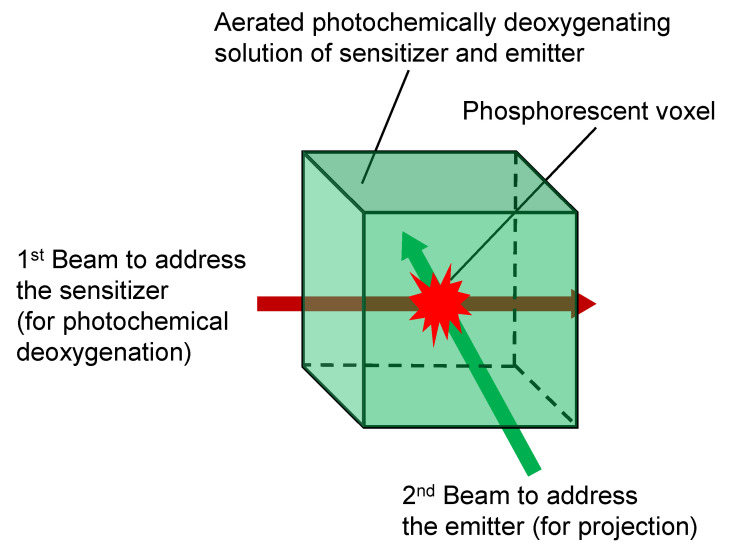
An illustration of the volumetric 3D display based on photoactivated phosphorescence.

In our previous work, we developed a 3D display prototype based on the phosphorescent complexes’ PAP [43]. Scheme 1 shows the PAP volumetric 3D display system we developed. The system includes a photochemically deoxygenated solvent and two phosphorescent complexes. One acts as the sensitizer and the other as the emitter. Under the irradiation of the first beam, the sensitizer absorbs photons and activates molecular oxygen (^3^O_2_). The photochemically-deoxygenating solvent scavenges the excited oxygen (^1^O_2_), forming a deoxygenated zone in the light path. Another wavelength is used to excite the emitter. At the intersection of the two beams, the emitter’s phosphorescence can be quickly photoactivated and a phosphorescent voxel is generated. The PAP 3D display system is simple to produce, features high-contrast images, and is easily observed with the naked eye.

The sensitizer and emitter are the key components in PAP 3D displays. These rules can determine sensitizer/emitter couples: (1) they have long-lived excited triplet states; (2) the emitter’s phosphorescence can be photoactivated in photochemically-deoxygenating solutions; (3) their absorption spectra are orthogonal to each other and can be independently addressed by two beams; (4) the sensitizer is not emissive in the visible region or not emissive at all; (5) the emitter is highly emissive in the visible region; (6) the visible emission of the emitter does not overlap with the absorption of the sensitizer. In accordance with these rules, the metal–porphyrin complexes Pt(II) tetraphenyltetrabenzoporphyrin (Pt(TPBP)) and Pt(II) octaethylporphyrin (Pt(OEP)) were used as the sensitizer and emitter and a PAP 3D display system with an emission wavelength of 645 nm was achieved. As the sensitizer in this display system, Pt(TPBP) phosphorescence is in the 710–850 nm region with a maximum of 770 nm. This visible red emission forms a background that lowers display quality. New sensitizers are still needed to eliminate the display system background.

To obtain bright 3D images, we hypothesized that the sensitizer could not reabsorb the emitter’s emission. In practice, it would be difficult for the sensitizer/emitter couple’s absorption and emission to be strictly orthogonal. If orthogonality is a prerequisite for bright 3D images, creating other single-color or multi-color 3D images is limited. In order to develop new luminescent materials for PAP 3D displays, it is essential to know whether reabsorption influences 3D image quality.

In this work, metal phthalocyanine complexes are the sensitizers and phosphorescent platinum compounds are the emitters in photochemically-deoxygenating solvents (sulfoxides and cyclic ureas) that can serve as luminescent materials for PAP 3D displays. The metal–phthalocyanine complexes we used had no emissions in the visible region. The phosphorescent–platinum compound and metal–phthalocyanine complex emissions overlapped. High-contrast 3D images were successfully generated using these luminescent materials as display mediums.

## 2. Materials and Methods

### 2.1. Materials

All starting materials were purchased from commercial sources and used as received unless stated otherwise. The dimethyl sulfoxide (DMSO), 1-methyl-2-pyrrolidinone (NMP), and tetramethylene sulfoxide (TMSO) used for imaging were of analytical grade. The DMSO, NMP, and TMSO used for photophysical measurements were of spectroscopic grade. 4-tertbutyl-phthalodinitrile was purchased from Frontier Scientific, Inc. (Philadelphia, PA, USA). 4-Bromonaphthalene anhydride, *N*-butylamine, and trimethylsilylacetylene were purchased from J&K Scientific Ltd. (Beijing, China). PdPrPc was synthesized according to the method specified in the literature [44].

### 2.2. Spectroscopic Characterization

^1^H and ^13^C NMR spectra were recorded on a Bruker Avance 500 FT-NMR spectrometer. HR-MS (high-resolution mass spectra) were obtained by a Thermo Scientific Q Exactive mass spectrometer, operated in heated electrospray ionization (HESI) mode and coupled with the Thermo Scientific UltiMate 3000 system. UV-Vis absorption spectra were recorded on a Thermo Scientific Evolution 201 UV-Visible Spectrophotometer. Photo-emission, excitation spectra, and lifetimes were recorded on an Edinburg spectrometer FLS-980 equipped with a Xe light source and an MCP-PMT detector in cooled housing (−20 °C), which covers a range of 200–870 nm. Lifetime data were analyzed with the F980 software package. Emission traces were recorded on an FLS-980 using the multiple scan mode and the shutter was set to always open. UV-portable UV lamps were used as light sources in the photoactivation processes. The optical power densities of these UV light sources were measured by a CEL-NP2000 optical power meter (Beijing CeauLight Technology Co., Ltd.). All measurements were performed at room temperature (RT).

### 2.3. Synthesis of PtNI

**(*N*-butyl-4-bromonaphthalimide)**: 4-Bromonaphthalene anhydride (1.40 g, 5.05 mmol), *N*-butylamine (0.74 g, 2 equiv.), and 20 mL acetic acid were added to a flask equipped with a condenser and magnetic stir bar. The mixture was heated to 120 °C overnight. After cooling to room temperature, a light-yellow solid was formed and filtered. The crude product was purified on a silica gel column (CH_2_Cl_2_: hexane = 1:2, *v*/*v*). ^1^H NMR (500 MHz, CDCl_3_): δ = 8.68–8.66 (d, 1H), δ = 8.59–8.56 (d, 1H), δ = 8.43–8.41 (d, 1H), δ = 8.06–8.04 (d, 1H), δ = 7.88–7.84 (t, 1H), δ = 4.21–4.17 (t, 2H), δ = 1.77–1.70 (m, 2H), δ = 1.52–1.42 (m, 2H), δ = 1.02–0.98 (t, 3H). ^13^C NMR (125 MHz, CDCl_3_): 163.63, 163.61, 133.19, 132.00, 131.19, 131.09, 130.63, 130.17, 129.01, 128.07, 123.19, 122.32, 40.39, 30.18, 20.38, 13.83.

**NI-C≡C-H**: *N*-Butyl-4-bromonaphthalimide (0.5 g, 1.5 mmol), Pd(PPh_3_)_2_Cl_2_ (21.0 mg, 0.03 mmol), PPh_3_ (16.0 mg, 0.06 mmol), and CuI (12.0 mg, 0.06 mmol) were added to a dry three-necked flask equipped with a magnetic stir bar. The flask was then evacuated and backfilled with nitrogen. This evacuation and backfill procedure was repeated three times. Then 10 mL dry THF, 10 mL triethylamine, and 0.6 mL trimethylsilylacetylene were added in a nitrogen atmosphere, respectively. The mixture was heated at 70 °C for 6 h in a nitrogen atmosphere. The reaction mixture was allowed to cool to room temperature, concentrated under reduced pressure, and then purified by column chromatography (silica gel, CH_2_Cl_2_: hexane = 1:1, *v*/*v*). The silyl group was removed by treating it with anhydrous potassium carbonate in methanol at room temperature. After removing the methanol under reduced pressure, the residue was dissolved in 30 mL dichloromethane and washed with water (2 × 25 mL). The organic layer was dried in anhydrous Na_2_SO_4_. The solvent was removed under reduced pressure, the crude product was purified with column chromatography (silica gel, CH_2_Cl_2_: ethyl acetate = 10:1, *v*/*v*), and a yellow powder was obtained. Yield: 100 mg, 24%. ^1^H NMR (500 MHz, CDCl_3_): δ = 8.70–8.68 (d, 1H), δ = 8.67–8.65 (d, 1H), δ = 8.57–8.55 (d, 1H), δ = 7.97–7.96 (d, 1H), δ = 7.87–7.84 (t, 1H), δ = 4.22–4.19 (t, 2H), δ = 3.76 (s, 1H), δ = 1.77–1.71 (m, 2H), δ = 1.51–1.44 (m, 2H), δ = 1.02–0.99 (t, 3H). ^13^C NMR (125 MHz, CDCl_3_): 163.97, 163.70, 132.19, 131.98, 131.69, 131.67, 130.16, 127.94, 127.71, 126.19, 123.03, 122.88, 86.46, 80.35, 40.38, 30.21, 20.40, 13.87.

**PtNI**: The complex PtNI was prepared according to the literature method [45]. Pt(dbbpy)Cl_2_ (50 mg, 0.09 mmol), CuI (5 mg, 0.09 mmol), and diisopropylamine (1.0 mL) were dissolved in CH_2_Cl_2_ (5 mL) and the mixture was stirred for 10 min. The mixture was purged with nitrogen and NI-C≡C-H (100 mg, 0.36 mmol) was added and stirred at room temperature for 24 h. The reaction mixture was evaporated to dryness. The residue was purified by column chromatography (silica gel, CH_2_Cl_2_: CH_3_OH = 150:1, *v*/*v*) to create a yellow powder (Yield: 55 mg, 60%). The product was further purified by slow diffusion of diethyl ether into its CH_2_Cl_2_ solution. Yield: 32 mg, 35%. ^1^H NMR (500 MHz, CDCl_3_): δ = 9.69–9.68 (d, 2H), δ = 9.16–6.13 (d, 2H), δ = 8.56–8.50 (m, 4H), δ = 8.05 (s, 2H), δ = 7.90–7.88 (d, 2H), δ = 7.69–7.67 (d, 1H), δ = 7.52–7.48 (m, 2H), δ = 4.20–4.16 (t, 4H), δ = 1.76–1.69 (m, 4H), δ = 1.53–1.40 (m, 22H), δ = 1.0–0.96 (t, 6H). HR-ESI-MS: *m*/*z*: 1015.3698 ([M+H^+^]^+^), 1037.3519 ([M+Na^+^]^+^).

### 2.4. Synthesis of PdBuPc

4-tertbutyl-phthalodinitrile (100 mg, 0.14 mmol) and Pd(OAc)_2_ (63 mg, 0.28 mmol) were added to a 25 mL flask containing 10 mL of NMP. The mixture was heated at 200 °C for 48 h under stirring. The reaction mixture was filtered and the filter residue was collected. The filter residue was dissolved in chloroform, purified by column chromatography on silica gel (using chloroform as an eluent), and then concentrated. Yield: 67%. HR-ESI-MS: *m*/*z*: 842.3126 ([M+H^+^]^+^).

### 2.5. General Methods for PAP 3D Images

A DMSO/NMP (TMSO) solution of PdPrPc (1 × 10^−5^ mol dm^−3^) (PdBuPc, 5 × 10^−6^ mol dm^−3^) and PtNI (1 × 10^−5^ mol dm^−3^ for Material 1 and 5 × 10^−6^ mol dm^−3^ for Material 2) were prepared and loaded into the imaging chamber. A 635 nm diode laser (OPD 10.4 mW cm^−2^) with a linear-shaped cross-section was used as the deoxygenation light source. The BenQ MH741 1920 × 1080 dpi projector was placed 18 cm away from the center of the imaging chamber. PowerPoint was used to draw figures and project the images. The figures’ color and the PowerPoint slides’ background were set to blue (440 nm, RGB (255, 0, 0), OPD 10.1 mW cm^−2^) and black, respectively. The laser beam was tilted inward at 30° for a better viewing angle. Images were acquired using a smartphone camera.

## 3. Results and Discussion

The phosphorescent complex PtNI was synthesized by reacting Pt(dbbpy)Cl_2_ with the ligand of NI-C≡C-H, purifying it by column chromatography and then recrystallization. Multinuclear NMR and high-resolution ESI mass spectrometry characterized PtNI. PtNI is soluble in DMSO, TMSO, and NMP. PdBuPc was synthesized by reacting the ligand of 4-tertbutyl-phthalodinitrile with Pd(OAc)_2_ at high temperatures in an NMP solution. The solubility of 4-tertbutyl-phthalodinitrile in NMP is poor and a long reaction time is needed. In dichloromethane, the absorption maximum of the Q-band of 4-tertbutyl-phthalodinitrile is 698 nm and the absorption maximum of the Q-band of PdBuPc is 660 nm. The reaction can be monitored by recording UV-Vis absorption spectra. PdBuPc has a high solubility in tetrahydrofuran (THF), chloroform, and dichloromethane but poor solubility in TMSO and NMP. High-resolution ESI mass spectrometry characterization confirmed the formation of PdBuPc.

**Figure 1:**
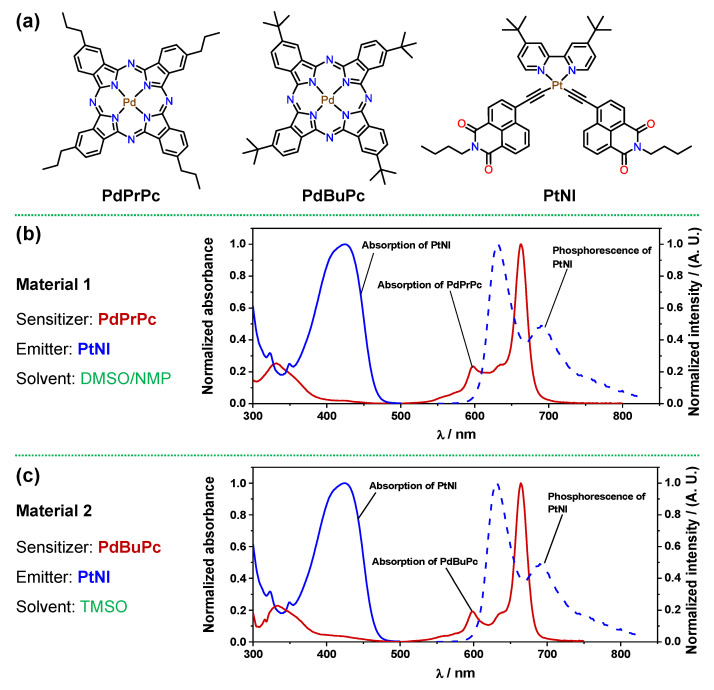
(**a**) Chemical structures of PdPrPc, PdBuPc, and PtNI. (**b**) Absorption and emission spectra of PdPrPc and PtNI in DMSO/NMP solutions. (**c**) Absorption and emission spectra of PdBuPc and PtNI in TMSO solutions.

According to the rules for selecting sensitizer/emitter couples for PAP 3D displays, metal–phthalocyanine complexes have a wide gap between the S_0_ → S_2_ Soret and the S_0_ → S_1_ Q-bands in the absorption spectra, which could be good sensitizer candidates for PAP 3D displays. Furthermore, they have a high quantum yield of singlet oxygen and their phosphorescent emissions are in the near-infrared region [46,47,48]. Here, two metal–phthalocyanine complexes, PdPrPc and PdBuPc, were selected as sensitizers. Figure 1 shows the chemical structures and absorption spectra of complexes PdPrPc and PdBuPc. PdPrPc and PdBuPc have similar absorption profiles in DMSO/NMP (Material 1) and TMSO (Material 2) solutions. PdPrPc in DMSO/NMP shows zero absorbance in the 450–515 nm region and weak absorption in the 400–450 nm region. PdBuPc in TMSO shows zero absorbance in the 470–515 nm region and weak absorption in the 450–470 nm region. In accordance with to the absorption profile of PdPrPc or PdBuPc, PtNI was selected as the emitter. As shown in Figure 1, the absorption and phosphorescence bands of PtNI are at 340–490 nm and 580–830 nm, with a maximum of 630 nm, respectively. The 450–490 nm region provides a window for photo-excitation of PtNI in blue light. There is an overlap between the emission spectrum of PtNI and the absorption spectra of PdPrPc or PdBuPc. The gap between the emission maximum of PtNI and the absorption maximum of PdPrPc or PdBuPc is approximately 32 nm. DMSO, NMP, and TMSO are photochemically-deoxygenating solvents that have also been demonstrated [40,41,42]. According to the solubility of PdPrPc or PdBuPc, DMSO/NMP (*v*:*v* = 1:1) was used as the solvent for PdPrPc/PtNI and TMSO for PdPrPc/PtNI.

**Figure 2:**
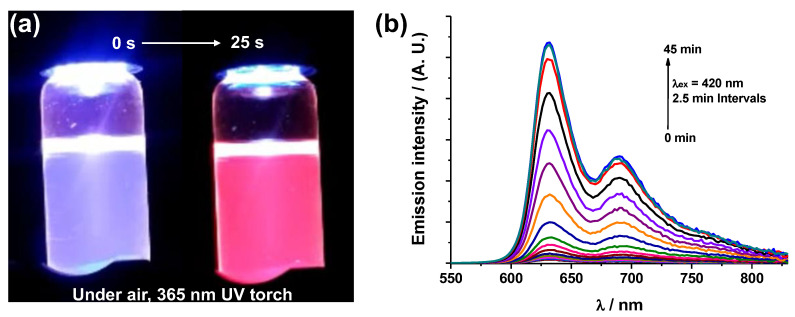
(**a**) Switched-on emission from an aerated DMSO solution of PtNI after being irradiated with a 365 nm UV torch. (**b**) Emission traces of an aerated DMSO solution of PtNI (1 × 10^−5^ mol dm^−3^) upon continuous 420 nm excitation at 298 K.

The phosphorescence of emitters for PAP 3D displays must be photoactivated in photochemically-deoxygenated solvents. PtNI is a room-temperature phosphorescent complex with a quantum yield of 21.5% and a lifetime of 124 μs in 2-methyltetrahydrofuran at room temperature under argon [45]. This complex may be an emitter candidate for PAP 3D displays. The phosphorescence lifetimes of PtNI in nitrogen-bubbled and photo-deoxygenated DMSO solutions were 80 and 91 μs, respectively, indicating that PtNI phosphorescence could be completely quenched by molecular oxygen in the air. As shown in Figure 2a, PtNI is virtually nonemissive in DMSO solution under air at ambient temperatures. Upon continuous irradiation with a 365 nm UV torch (optical power density (OPD) ~75 mW cm^−2^), a red emission developed within 25 s. Shaking the solution in the air extinguished the red emission. The emission spectra of an aerated DMSO solution of PtNI during photoactivation processes were recorded on a spectrofluorometer upon excitation at 420 nm (Figure 2b). An emission band with a peak maximum at 630 nm gradually developed and reached its maximum after ~45 min. These results demonstrated that PtNI phosphorescence could be photoactivated in a DMSO solution and that the light beam could address its on-off states.

**Figure 3:**
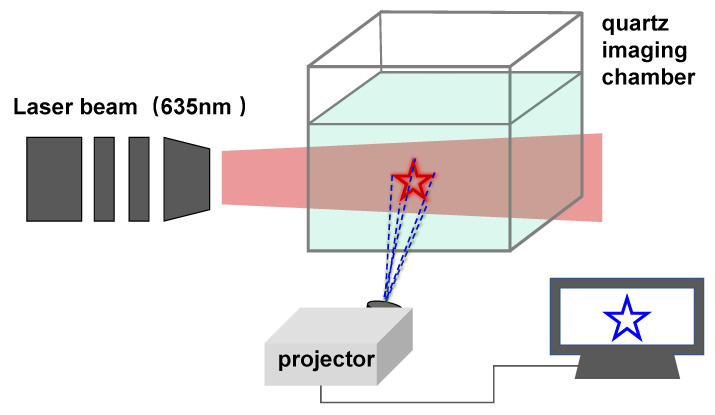
Schematic of the PAP 3D display system structure.

We designed a PAP 3D display system to produce 3D images. As shown in Figure 3, the graphics engine contains a personal computer to control a digital light-processing (DLP) projector. This projector can output two-dimensional (2D) images with a 440 nm light, a 635 nm diode laser with a linear-shaped cross-section, and a quartz chamber containing a solution of sensitizer and emitter. The 635 nm linear laser travels through the solution to form a photochemicallydeoxygenated rectangular zone acting as a virtual ‘screen’. The 440 nm beam from the projector with cross-sections shaped by the computer excites PtNI in the deoxygenated ‘screen’ to provide visible images.

**Figure 4:**
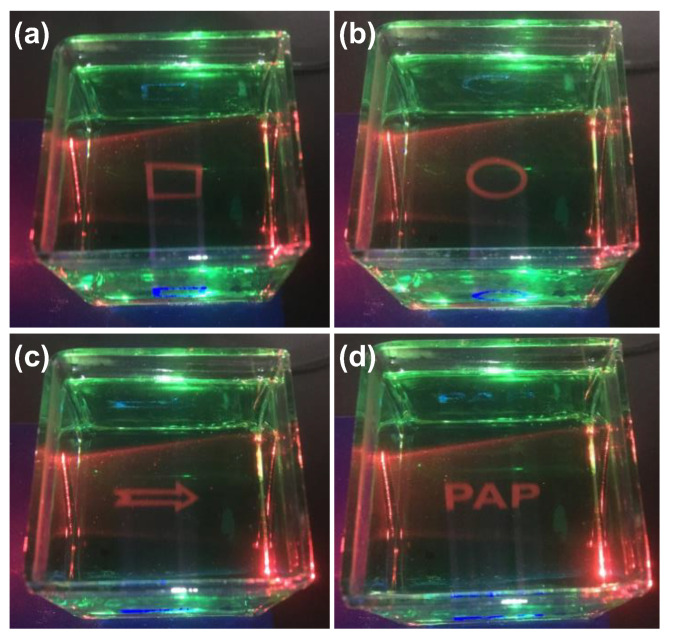
Snapshots of the PAP images inside a bulk PdPrPc/PtNI DMSO/NMP solution contained in a 5 × 5 × 5 cm^3^ cubic quartz chamber. (**a**) a square, (**b**) a cycle, (**c**) an arrow, (**d**) the letters ‘PAP’.

We set up a homemade PAP 3D display system. Initially, Material 1 was used as the display medium for 3D images. A stock solution of PdPrPc and PtNI mixture dissolved in DMSO/NMP with a concentration of 1.0 × 10^−5^ and 1.0 × 10^−5^ mol dm^−3^, respectively, was prepared. The solution was loaded into a quartz chamber (5 × 5 × 5 cm^3^). Firstly, under the illumination of a red linear-shaped laser, a deoxygenated zone formed in the solution and this zone defined the depth like a ‘screen’. Secondly, high-resolution images, such as a square, a cycle, an arrow, and the letters ‘PAP’, were projected in the vertical direction of the red beam. Geometric figures and letters were generated on the ‘screen’ (Figure 4). The images displayed inside the solution were legible under ambient light and without additional eyeglasses or filters.

**Figure 5:**
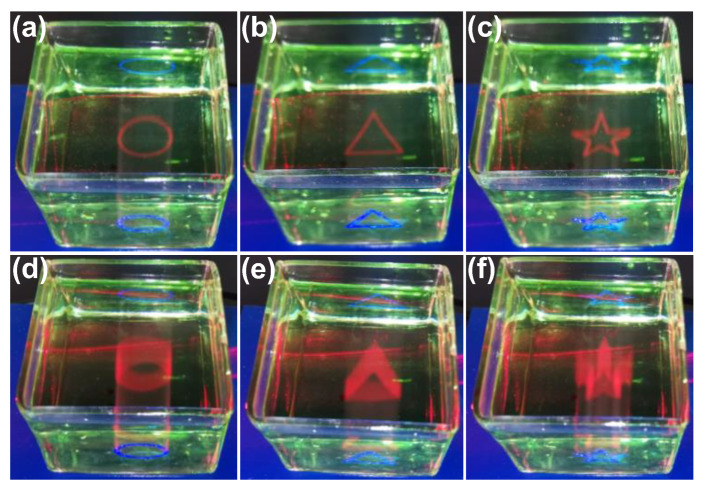
(**a**–**c**) Snapshots of the PAP images; (**d**–**f**) snapshots of spatially defined PAP 3D cylinders with various shapes inside a bulk PdBuPc/PtNI TMSO solution contained in a 5 × 5 × 5 cm^3^ cubic quartz chamber.

Afterward, we tested the PdBuPc/PtNI couple to see if the PAP display system functioned. A stock TMSO solution of PdBuPc and PtNI with a concentration of 5.0 × 10^−6^ and 5.0 × 10^−6^ mol dm^−3^ was prepared and loaded into a quartz chamber (5 × 5 × 5 cm^3^). 3D images of the cycle, triangle, and pentagram were projected using the PdPrPc/PtNI method (Figure 5a–c). In cylinder geometry, PAP 3D images can be projected by increasing the thickness of the deoxygenated zone. Figure 5d–f shows a spatially-defined cylinder, triangular prism, and pentagram. This display system had no background from PdBuPc emission and the images were bright under ambient light.

We successfully constructed a PAP 3D display prototype using both materials as luminescent mediums. These display systems generated 3D images with high brightness and contrast. PdPrPc and PdBuPc had no emissions in the visible region and no background was formed. These conditions increased image contrast. Although the phosphorescence of PtNI and the absorption of PdPrPc or PdBuPc overlapped, the reabsorption did not influence the 3D images’ brightness. In this experimental setup, we used a 440 nm wavelength (from the projector) to irradiate PtNI. PdPrPc and PdBuPc have weak absorption at this wavelength. In turn, the photoactivation of PtNI phosphorescence in the light path at 440 nm accelerates. Controlling the 440 nm light power can address the adverse effect on display quality. PdPrPc and PdBuPc are insoluble in DMSO and their solubility is poor in NMP or TMSO. Display quality and other metal phthalocyanine complexes with good solubility that would otherwise benefit the display system are adversely affected.

## 4. Conclusions

In summary, we developed two luminescent materials for PAP 3D displays. We constructed a PAP 3D display prototype and generated high-contrast 3D images using both materials as display mediums. The background was eliminated and the image contrast was increased using metal–phthalocyanine complexes with no emission in the visible region as sensitizers. Although there was a partial overlap between emitter emission and sensitizer absorption, high-quality 3D images were generated. This finding can guide the development of new luminescent materials for PAP 3D displays and encourage research into multi-colored PAP 3D displays.

## Data Availability

Not applicable.
